# Trends in scientific activity addressing transmissible spongiform encephalopathies: a bibliometric study covering the period 1973–2002

**DOI:** 10.1186/1471-2458-6-245

**Published:** 2006-10-06

**Authors:** Elías Sanz-Casado, Margarita Ramírez-de Santa Pau, Carlos A Suárez-Balseiro, Isabel Iribarren-Maestro, Jesús de Pedro-Cuesta

**Affiliations:** 1 Laboratory of Information Metric Studies (LEMI) – Library Science and Documentation Department. Carlos III University of Madrid. Madrid 126, 28903 Getafe, Spain; 2 Spanish Network for Cooperative Research in Neurological Diseases (CIEN) and Spanish Network for Cooperative Research in Epidemiology and Public Health (RCESP). Carlos III Institute of Health. Sinesio Delgado 6, 28029 Madrid, Spain; 3 Observatorio de Estudios Relacionados con la Información (OERI). School of Information Science and Technologies. Puerto Rico University, Recinto de Rio Piedras, Puerto Rico; 4 Applied Epidemiology Department. National Center of Epidemiology. Carlos III Institute of Health. Sinesio Delgado 6, 28029 Madrid, Spain

## Abstract

**Background:**

The purpose of this study is to analyse the trends in scientific research on transmissible spongiform encephalopathies by applying bibliometric tools to the scientific literature published between 1973 and 2002.

**Methods:**

The data for the study were obtained from Medline database, in order to determine the volume of scientific output in the above period, the countries involved, the type of document and the trends in the subject matters addressed. The period 1973–2002 was divided in three sub-periods.

**Results:**

We observed a significant growth in scientific production. The percentage of increase is 871.7 from 1973 to 2002. This is more evident since 1991 and particularly in the 1996–2001 period. The countries found to have the highest output were the United States, the United Kingdom, Japan, France and Germany. The evolution in the subject matters was almost constant in the three sub-periods in which the study was divided. In the first and second sub-periods, the subject matters of greatest interest were more general, i.e Nervous system or Nervous system diseases, Creutzfeldt-Jakob disease, Scrapie, and Chemicals and Drugs, but in the last sub-period, some changes were observed because the Prion-related matters had the greatest presence.

Collaboration among authors is small from 1973 to 1992, but increases notably in the third sub-period, and also the number of authors and clusters formed. Some of the authors, like Gajdusek or Prusiner, appear in the whole period.

**Conclusion:**

The study reveals a very high increase in scientific production. It is related also with the beginnings of research on bovine spongiform encephalopathy and variant Creutzfeldt-Jakob disease, with the establishment of progressive collaboration relationships and a reflection of public health concerns about this problem.

## Background

Prion diseases or transmissible spongiform encephalopathies (TSEs) encompass a series of pathologies that affect both animals and humans. They are characterised by their prolonged incubation period and are transmissible in laboratory animals. Moreover, degenerative lesions in the nervous system are marked by vacuolation of brain tissue, which may prove to have a spongiform appearance under the microscope [1]. Two German pathologists, Creutzfeldt and Jakob, were first to describe the disease which now bears their name when, in the early 1920s, they published an account of some patients with a complex neurological syndrome that progressed rapidly and could not be identified with any of the diagnostic entities known until then. The description of the transmissibility of another of these diseases -kuru- generated the term "slow viruses", on the assumption they were caused by certain unconventional viruses or pathogenic agents. Subsequently, Prusiner was to call the pathogenic agent a prion (proteinaceous infectious particle), an altered structural form of a protein found in the central nervous system, lymphatic system and neuromuscular junctions [2]. In 1997, he was awarded the Nobel Prize in recognition of the enormous biological implications of his studies. The importance and current relevance of these topics in public health is connected with the fact: that animal spongiform encephalopathies have, as explained below, crossed into the human species after prolonged dietary exposure by millions of persons; and that there is a precedent of a pandemic of these diseases, albeit in a small human population, i.e., kuru among the Fore people of Papua-New Guinea.

From a historical stance, the first known spongiform encephalopathies were animal, beginning with the description of scrapie in the 18^th ^century. Its transmission was proved experimentally in 1936 [3]. Scrapie occurs in sheep and goats worldwide with the sole exceptions of Australia and New Zealand. It appears from the age of two years onwards. A number of hypotheses exist as to its mode of transmission, linked to the existence of a gene that regulates susceptibility to the prion. To date, however, there is no evidence of it having been transmitted to man. Much more recently, other transmissible spongiform encephalopathies have been described in animals, including mink and the *Cervidae *(Chronic Wasting Disease in North American mule deer and elk). The bovine spongiform encephalopathy (BSE) that appeared in bovine cattle in the United Kingdom (UK) in 1985 and, since the 1990s, in some European Union countries is very recent. It affects cows aged 20 months to 18 years, though the highest incidence occurs in animals aged 2 to 8 years. The most plausible hypothesis is that the disease is triggered when meat-based feed containing scrapie-infected sheep offal are incorporated into animal feed. Transmissible spongiform encephalopathies are found in domestic animals (cats) and ungulates in UK zoos, and are also associated with consumption of infected meat-based animal feed.

In humans, the description of spongiform encephalopathies begins with Creutzfeldt-Jakob disease (CJD), identified in the 1920s. Essentially, four types or modalities have been described, namely, sporadic, iatrogenic or accidental, familial and variant. Approximately, 80%–90% of CJD cases are **sporadic**, are distributed worldwide and affect patients in the 18 to 80-and-over age range with an elevated incidence among adults aged 50–70 years. Incidence stands at around one case per million population per year. **Iatrogenic **or **accidental **CJD is caused by contamination in medical operations, such as cornea transplants, neurosurgical procedures, hormone treatments or dural implants with cadaveric tissue. **Familial **or **genetic **CJD affects 5%–15% of cases and is produced by mutations in the gene of the prionic protein located on chromosome 20. In general, it is similar in presentation to sporadic CJD but sometimes has type-specific features. **Variant **CJD (vCJD) was identified in 1996 [4] and has different characteristics to sporadic CJD. Currently, vCJD is causally attributed to ingestion of BSE-infected bovine tissue, which implies a jump of the species barrier, though the precise pathogenic mechanism is not known [5].

**Kuru**, described in 1957 by Gajdusek, affected the Fore people of Papua-New Guinea, particularly children, adolescents and women. Its origin is linked to cannibalism-related funerary practices that were ceased in the 1950s. The disease is now extinct. In the 1960s, the lesions caused by kuru, CJD and scrapie were discovered to be similar. Since then, other very infrequent non-CJD-genetic-type spongiform encephalopathies have been described, though this tends more to their molecular affiliation than it does to that of their clinical-expression: 1) **Gerstmann-Straussler-Scheinker Syndrome **(GSS) is caused by a mutation in codon 102 and other alterations in codons 105, 145 and 117; and 2) **Fatal familial insomnia **(FFI) is characterised by a mutation in codon 178, though other genetic disorders have also been described. In the last two years, descriptions of a case of blood-transmitted vCJD and possible atypical forms of these diseases transmitted in the same manner are posing a challenge for interpretation of data and the drawing-up of prognoses [6,7]. Thus, follow-up of research activity based on analysis of scientific literature is particularly illustrative of the yield from investment in new research topics.

The aim of this paper was to analyse the trend in research on transmissible spongiform encephalopathies through application of bibliometric tools to scientific literature published from 1973 to 2002. Based on the application of these techniques, we sought to ascertain the scientific production published in this period, the countries responsible, the trend in research topics, and the participant authors.

## Methods

Data for the study was obtained from Medline^©^, via the online PubMed^© ^service provided by the National Library of Medicine (NLM)[8]. The date of search was March 24^th^, 2004. For information-retrieval purposes, we consulted the controlled vocabulary (MESH) used by the NLM to index PubMed^© ^contents. The search strategy adopted was as follows:

encephalopathy, bovine spongiform [MESH] OR creutzfeldt-jakob syndrome [MESH] OR gerstmann-straussler-scheinker disease [MESH] OR insomnia, fatal familial [MESH] OR kuru [MESH] OR scrapie [MESH] OR prion diseases [MESH] OR prion [MESH] OR prion protein [MESH]

The accepted MESH term for Creutzfeldt-Jakob disease is "Creutzfeldt-Jakob syndrome", though the name has fallen into disuse in scientific circles.

This strategy located 7808 entries, which were then imported into Procite^©^, using the PubMed search module (PubMed Search) version 5.0 for Windows.

Once in Procite^©^, entries that were either duplicated or found to have incomplete information were deleted. The definitive database comprised 7800 entries, in which the information of bibliometric interest was quantitatively analysed. In those cases where information on the author's affiliation was not available, a search was made for the paper in question in the original source and the author's country of origin thus identified.

This study uses unidimensional- and multidimensional-type indicators. Whereas the former measure a single characteristic of documents published by researchers (scientific production, index of co-authorship, topic area of the publication), multidimensional indicators enable exploration and study of the interrelationships displayed in the documents. Analysis of co-occurrence of terms was used for a quantitative approach to the content structure of the publications, taking into account the frequency and strength of the links established. To obtain these indicators, statistical techniques of multivariate analysis, the so-called interdependence techniques, were used. In this particular case, multidimensional scaling (MDS), often used in bibliometric research on biomedical fields [9-12], and correspondence factorial analysis, were applied to explore collaboration among authors and the trend in the areas of research in which they worked.

For the study of the relationship between topic categories, we chose to use only those terms specified by MESH as indicators of the core content of the document published. On the basis of such terms we then consulted with experts, in order to standardise and, in some cases, add these for greater ease of reading.

All statistical analyses were performed using the *SPSS^® ^*10.1 and *Microsoft Excel*^® ^2003 computer software programmes. The study matrices were drawn up and the MDS-based results depicted using BibExcel, a freeware tool for bibliometric analysis developed by Olle Persson et al. at Umeå University [13].

## Results

### Scientific production

Table 1 shows percentage variations on the baseline value (corresponding to 1973) with respect to documents published over the three decades analysed. As can be seen, growth in scientific production was greatest in the last decade (1993–2002) with two major peaks in 1991 and 1996. In 1991, the increase on the preceding year (1990) was 83.3%, and in 1996 the increase on 1995 was 159%.

### Geographical distribution of scientific production

The countries with greatest scientific production in this field are ranked in Table 2 according to the respective trends in their absolute and percentage values over the three periods analysed. To render the depiction of data clearer, we only considered percentages of those countries that surpassed 1% of documents published in each of the periods. The country that maintained the highest scientific production over the three periods was the United States (USA), accounting for 40.2% and 41.1% of all documents published in the first two decades, with this percentage decreasing to 26.7% in the third period. In this last decade, the relative weight of the United States declined, though in absolute values its publishing activity registered a 134.8% increase over the previous decade.

The second leading country in number of documents published over the three decades was the United Kingdom, with a mean percentage slightly higher than 20%. Unlike the USA, in the third period the UK maintained its weight with respect to world production.

Japan and France were countries that registered a considerable level of research activity. Although Japan's relative weight declined in the third period, its scientific production rose, registering a 2.4-fold increase in absolute values vis-à-vis the second decade. France improved its standing, moving into third place in the 1993–2002 period. Attention must also be drawn to the case of Switzerland, whose scientific production in the last ten-year period accounted for 4.6% of all documents published.

### Characteristics of scientific production: type of document

The type of scientific document most used to disseminate research was the journal article or paper in 81.4% of cases, followed by the letter in close on 10% of cases, and news in 5.2% of cases (Table 3).

### Topic structure of research activity

Topics which proved of greatest interest to researchers and, in addition, appeared in more than 1% of documents are shown in Table 4 [see Additional file 1]. A number of different topics were normally assigned to each document, with the result that the total percentage exceeds 100%.

In the first period (1973–1982), the research topics of greatest interest were related to the Nervous system or Nervous system diseases (44.4%), with some aspects linked to Creutzfeldt-Jakob disease (27.6%), Scrapie (17.4%), and Chemicals and Drugs (16.9%). It should be noted, however, that in this period, the documents selected were directly related to some aspect connected with Slow Virus Diseases (SVD) in 5.9%, prions in 5.6% and kuru in 3.6% of cases.

During the second period (1983–92), the topics that appeared most frequently were the same as those in the preceding period, though there was a considerable increase in terms of both absolute and percentage values. Topics that accounted for the highest percentage were Nervous system or Nervous system diseases (39.6%), and Chemicals and Drugs (32.3%), while documents linked directly to Creutzfeldt-Jakob disease experienced a decline in their relative weight with respect to the previous period, to 22.4%, but a rise in their absolute value (301 versus 180 documents in the 1973–1982 period). Other topics that increased their presence with respect to the preceding period were Scrapie, in 19.1% of documents, and to a notable degree, prions, which increased their presence to 13.3% of publications in this decade.

Similarly noteworthy in this period was the appearance of topics such as Bovine Spongiform Encephalopathy (BSE) (2.5%) and Gerstmann-Straussler-Scheinker Disease (GSSD) (1.2%), as well as the absence of SVD and kuru from the topics that registered a percentage in excess of 1%.

In the last period (1993–2002), some changes were observed in the topics that appeared most frequently, inasmuch as prion-related matters now had the greatest presence (34.3%), followed by Chemicals and Drugs (28.8%), and Nervous system and Nervous system diseases (21.8%). High percentages were also registered for Biological Sciences and Bovine Spongiform Encephalopathy (BSE). This last topic, which had already appeared in the previous period (1983–1992) -though with only 34 documents (2.5%)- underwent a 13-fold increase in terms of absolute values in this period, rising to 458 documents and thus accounting for 10.7% of all documents published.

Insofar as documents relating to Creutzfeldt-Jakob disease were concerned, these lost relative weight with respect to the preceding periods, with only 17.1% addressing some aspect linked to this disease, though absolute values increased 2.5 times on the previous period. The same happened with Scrapie: its percentage value fell (8.9%) but its absolute value rose in terms of the number of documents vis-à-vis the previous decade (383 versus 257).

### Scientific collaboration

By focusing on analysing the authors that signed the documents, this study enabled the collaboration established by these researchers to be ascertained. Tables 5, 6 and 7 list all the authors constituting the clusters observed in each of the periods analysed. To determine the most productive clusters, we only considered authors credited with more than 1% of documents produced in collaboration.

In the first period (1973–1982), shown in Table 5 [see Additional file 2], a total of 8 clusters will be observed. The largest of these (C1) was made up of the researchers, Gajdusek DC, Brown P, Cathala F, Gibbs CJJr, Asher DM, Court L, Masters CL, Moreau Dubois MC and Rohwer RG, the first four of whom -Gajdusek DC in particular- were responsible for setting up the greatest number of collaborations. The second cluster in terms of size (C2) was formed by Kimberlin RH, Millson GC, Marsh RF, Hunter GD, Collis SC, Hanson RP and Walker CA. Another of the larger-sized clusters (C3) comprised the authors, Prusiner SB, Cochran SP, Groth DF, McKinley MP and Baringer JR, with Prusiner SB being the most productive.

The remaining clusters were very small, some formed by only two authors, such as the case of Manuelidis E. and Manuelidis L. (C7), as well as that grouping Bert J and Tamalet J. (C8).

In the second decade (1983–92), the composition of the different clusters was as shown in Table 6 [see Additional file 3]. Like the previous period, 8 clusters were again obtained, though in most cases the constituent authors had changed. The first of these (C1) continued to be the most productive and was formed by Gajdusek DC, Gibbs CJJr, Cathala F, Brown P, Chatelain J, Asher DM, Goldfarb LG, Liberski PP, Pocchiari M and Yanagihara R. The first four authors had also appeared together in the preceding period.

Prusiner SB, Bendheim PE, DeArmond SJ, McKinley MP, Bolton DC, Westaway D participated in the second cluster (C2). In this case, only two authors coincided with the previous period, namely, Prusiner SB and McKinley MP, with the former continuing to be the most productive. As before, there was also a small cluster (C7) in this period made up of Manuelidis EE and Manuelidis L.

As a result of a split in a cluster that had existed in the previous period, two new clusters came into being. The first of these (C3) was formed by Carp RI, Diringer H, Kascsak RJ, Kimberlin RH, Wisniewski HM, Rubenstein R, Walter CA and Merz PA. Kimberlin RH and Walter CA, who had coincided in the previous period, proved to be the researchers who now collaborated most intensely. The other cluster (C4) was formed by Hope J, Foster JD, Dickinson AG, Hunter N, Marsh RF and Somerville RA. Here, the researchers Hunter N and Marsh RF had participated in the same cluster in the preceding period.

Another of the newly formed clusters (C5) was made up of Tateishi J, Kitamoto T and Dohura K, only the first of whom had appeared in the previous period.

In the last of the periods reviewed (1993–2002) there were important changes with respect to the previous two, both as regards the number of authors and as regards the number of clusters. In this decade, not only did the number of authors rise notably as a result of more collaborations being established among them, but the number of clusters also increased to 12. Table 7 [see Additional file 4] shows authors' clusters of this.

Part of the researchers in this period continued with their scientific work undertaken in the previous two decades, as in the cases of Gajdusek DC and Brown P, and Prusiner SB, who continued to lead two of the most productive clusters, C1 and C2 respectively, as well as setting up collaborations with other researchers. Similarly, the cluster formed by Kitamoto T and Tateishi J (C11) also continued in this period but without maintaining important collaborations with other authors.

It should be noted that several clusters appeared for the first time in this decade. Among these, special mention must be made of those formed by: Zerr L, Kretzschmar HA and Poser S, among others (C4); Will RG, Pocchiari M, Ironside JW and Zeidler M (C6); Aguzzi A, Brandner, S, Klein MA and Weissmann C (C9); and, lastly, Gambetti P, Sy MS, O'Rourke KI, Parchi P, Petersen RB, Capellari S and Wong BS (C8).

### Relationship between clusters and the topics published by them

**Figure 1:**
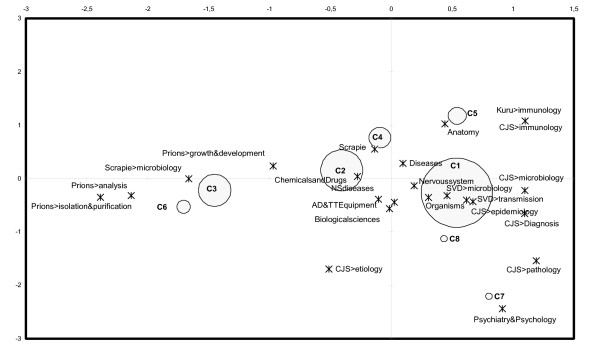
**Author's clusters and their position in the research subjects-matters space, 1973–1982. **Circles represent author's clusters, and their size show the cluster's productivity. They are placed next to their main subjects of research. The asterisks show the subject matters, related to the angle and proximity to axis.

**Figure 2:**
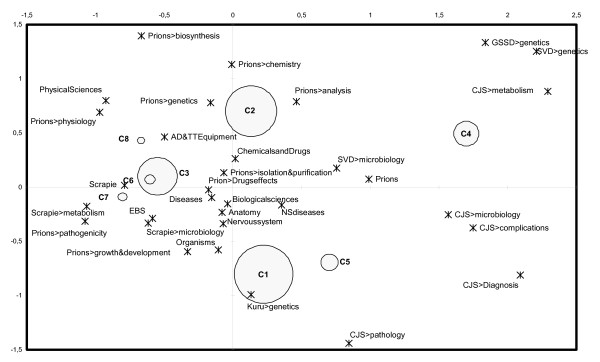
**Author's clusters and their position in the research subjects-matters space, 1983–1992**. Circles represent author's clusters, and their size show the cluster's productivity. They are placed next to their main subjects of research. The asterisks show the subject matters, related to the angle and proximity to axis.

**Figure 3:**
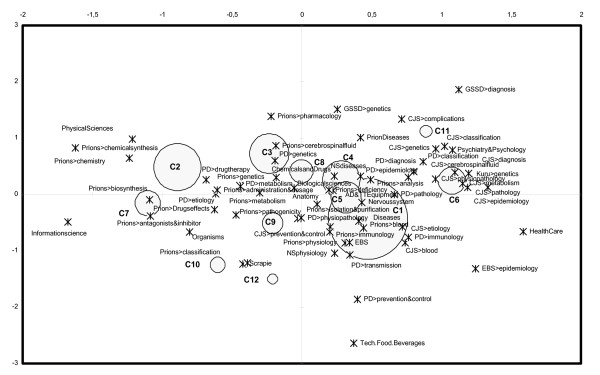
**Author's clusters and their position in the research subjects-matters space, 1993–2002**. Circles represent author's clusters, and their size show the cluster's productivity. They are placed next to their main subjects of research. The asterisks show the subject matters, related to the angle and proximity to axis.

Analysis of the topics on which the cluster researchers published in the three periods reviewed is depicted in Figures 1, 2 and 3, using the technique of correspondence factorial analysis. Shown in the centre of the chart are the core topics on which most of the clusters published, whilst in the peripheral areas are those addressed specifically by some of them.

The decade from 1973 to 1982 is shown in Figure 1, with a number of clusters appearing in the centre of the plot, as well as the topics most published in the period (Scrapie, Nervous system, Chemical & Drugs, Diseases or Nervous-System-Diseases). On the right is: C1, the most productive cluster of the period, formed by Gajdusek DC, Brown P and Cathala F, among others, which published on several different topics, ranging from those in the centre to Organisms and CJD-epidemiology; and C5, formed by Field EJ, Narang HK and Shenton BK, which focused its research on Anatomy, Kuru-immunology and CJD-immunology. Also in the centre but to the left are other clusters: C2, comprising Kimberlin RH, Millson GC and Marsh RF, which published on Scrapie, Chemical & Drugs and Nervous system diseases; and C4, formed by Fraser H, Dickinson AG and Outram GW, which published mostly on Scrapie.

Located further from the centre are a series of clusters: two on the left-hand side, namely, C3, with Prusiner SB, Cochran SP and Groth DF, which published specifically on Prions-analysis, Prions-isolation & purification, and C6, formed by Hadlow WJ, Eklund CM and Race RE, and specialised in the same research topics as C3; and another on the lower right-hand side (C7), formed by Manuelidis EE and Manuelidis L, which basically published on topics linked to Psychiatry & Psychology and CJD-pathology.

Shown in Figure 2 are the topics on which the clusters published during the period 1983–1992. From the figure, it will be seen that most of the clusters tended to migrate towards the centre of the plot. Lying in the lower right quadrant is C1, the cluster led by Gajdusek DC, which continued to be the most productive and, apart from the core topics, also published on Kuru-genetics and CJD-pathology. Nearby is C5, a cluster formed in this case by Tateishi J, Kitamoto T and Dohura K, which, in addition to the topics in the central area, published on CJD-pathology.

Cluster C2, situated in the upper quadrant and led by Prusiner SB, continued to publish on the core topics, as well as on Prions-analysis, Prions-genetics, Prions-isolation & purification. In the same quadrant but on the periphery of the plot is C4, formed by Hope J, Foster JD and Dickinson AG among others, which specifically published on CJD-metabolism, SVD-genetics (though many papers had already stopped using this term) and GSSD-genetics.

Situated to the right of the chart are 4 clusters, two of which overlap, C3 and C6: the former was the more productive and was formed by Carp RI, Diringer H and Kascsak RJ, among others, and the latter was formed by Bruce ME and Fraser H. Both of these clusters published on the core topics, and specifically on Scrapie. Cluster C7, which lies near the other two and was formed by Manuelidis EE and Manuelidis L, shared a number of topic areas with those in the centre and, in addition, published on Scrapie and BSE.

In the last period (1993–2002), the trend in the previous two periods emerges, with an increase both in the number of clusters participating and in the topics on which they published (Figure 3). As in the preceding period, most of the clusters are seen to occupy a central position, due to the fact that they share many of the core research topics which lie in this area and are practically the same as those in the previous periods (Chemical & Drugs, Nervous-system, Biological-sciences, Diseases, Nervous-systems-diseases and Anatomy).

Lying in the right-hand quadrant of the plot, near the centre and almost superimposed are three clusters, namely: C1, which continued to be the most productive and in which Gajdusek DC and Brown P still participated; C5, formed by Laplanche JL, Hauw JJ and Dormont D among others; and C4, with Kretzschmar HA, Zerr I and Groschup MH. These three clusters maintained a high rate of research activity in most of the core topic areas, though C1 was more closely linked to BSE and a number of prion-related topics (Prions-immunology, Prions-analysis, Prions-isolation & purification or Prions-blood), whilst C4 focused on Chemical & Drugs.

In the right-hand area of the chart, away from the centre, are two more clusters. These are: C6, with Will RG, Pocchiari M and Ironside JW, which published with great intensity on some of the core topics, as well as on neighbouring subjects such as those related to Creutzfeldt-Jakob disease (CJD-diagnosis, CJD-epidemiology, CJD-pathology or CJD-genetics) and Kuru (Kuru-genetics); and C11, formed by Kitamoto T and Tateishi J, which published on CJD-genetics, GSSD-diagnosis, Psychiatry & Psychology.

Situated at the centre of the plot are the following 3 clusters: C8, formed by Gambetti P, Sy MS and O'Rourke KI, among others; C3, formed by Bugiani O, Tagliavini F and Salmona M; and C9, formed by Aguzzi A, Brandner S and Klein MA. Although these 3 clusters essentially published on the core topics, C3 was also active in the area of Prions-diseases-genetics and Prions-pharmacology, and C9 in Scrapie.

There are also several clusters on the left, the most important of them, in order of productivity being C2, in which Prusiner SB continued participating and which, aside from the core topics, published on Prions-chemistry, Prions-genetics and Prions-chemical-synthesis. Lying near this cluster is C7, formed by Caughey B, Priola SA and Horiuchi M, which published specifically on Scrapie, Organisms, Prions-drugs-effects, and Prions-antagonists and inhibitors.

Finally, appearing at lower left are another two smaller clusters: C10, formed by Hunter N, Hope J and Goldmann W; and C12, formed by McConnell I, Fraser H and Taylor DM. Both had a highly active output in terms of Scrapie and BSE, though the former also published on Organisms.

## Discussion

TSEs acquired relevance in scientific thinking in the 1960s. Subsequently, Gajdusek was awarded the Nobel Prize for demonstrating the transmissibility of kuru, a description which, in the previous decade, had formed part of the series of nervous system diseases described following the spread of US troops across the Pacific area after the Second World War. Examples of this are the foci of motor neurone disease in Japan and other islands, or the dementia-parkinsonism-motor neurone disease complex on the Island of Guam. Although key etiological factors, such as the food-related toxicity of *cynca circinalis *in the latter instance, constituted important revelations in human biology with impact on neurotoxicological research, none received anything like the attention given to spongiform encephalopathies, which has only been tempered by the favourable trend in the vCJD epidemic in the United Kingdom.

The results of this study show the specific trend in scientific output on the topics studied, with times when the pace of publication rose notably, e.g., in 1991 and 1996 the number of documents rose by 174% and 361.5% respectively vis-à-vis the 1973 base-year figure. These high publication rates coincide with the years in which research into these subjects benefited from special support. Specifically, BSE research has enjoyed the financial backing of the European Union since 1990, within the framework of its research and technological development programmes. Though doubtless related to the British government's decision in the preceding years to start eliminating bovine brain and marrow from the food chain and introduce human spongiform encephalopathy surveillance, this support nevertheless dates, above all, from the announcement made by the British authorities on 20 March 1996 that a new variant of Creutzfeldt-Jakob disease had been detected. It was at this point that the Commission approved a TSE research action plan that took into account the recommendations of the reports issued by the Weissman Group, set up in April 1996, and the Multidisciplinary Scientific Committee, as well as the results of ongoing research at a national and EU level. In this regard, the results obtained in this study clearly respond, in part, to the scientific effort made by the European Union in the 1990s and new discoveries in this field linking BSE to Creutzfeldt-Jakob disease.

The country that registers the greatest scientific production across the three periods is the United States and, despite the fact that its relative weight declined in the last decade, its absolute values remained very high, with its scientific activity increasing by almost 135% with respect to the preceding decade. It was the greater scientific activity of the other countries that underlay the relative decline of the United States.

The second leading country in terms of the number of documents published is the United Kingdom. The United Kingdom's intense scientific activity is connected with the appearance of BSE, with close on 10% of the two hundred thousand reported BSE cases being diagnosed in UK cattle [14]. Almost all the vCJD victims, some 170 by the end of 2005, had occurred in this country, with diagnosis of this disease outside the British Isles being exceptional, save in the case of France. Another country with a high degree of scientific activity is Japan. Incidence of BSE in their cattle stocks might possibly account for the great scientific activity in other countries, such as France and Switzerland, which notably raised its profile in the third period, occupying sixth place.

The most popular type of document for disseminating research results proved to be the scientific paper, a finding in line with other bibliometric studies conducted in the field of health sciences [15].

The selection of data source is one of the most important decisions for bibliometricians. Although there are other databases available, Medline is widely used and currently considered the best bibliographic source in biomedicine. Therefore, the search performed through PubMed services could be considered complete enough for the purposes of this study. Yet our study methodology implies a bias *per se*, inasmuch as it relies on the Medline database in which documents are mainly sourced from the indexing of scientific journals. However, it could be noted that the use of Medline database is not exempt of problems. In fact, the outputs depend on how the databases are constructed and structured. Some authors have brought to discussion the bias of Medline. For instance, Ojasoo et al. mentioned the existence of quirks in Medline indexing of publication types in clinical medicine. According to this fact, they have questioned the consistency of indexing procedures and also the rationale for the database's choice of descriptors. They suggest observed trends might not always reflect true publication trends [16]. The normalization issue is another problem identified when using Medline for bibliometric studies. This problem has been remarked, specifically for Spanish names in the Authors and Address fields [17,18].

Accessing other sources would enormously hinder the handling and processing of any information retrieved, which would be less homogenous but would nonetheless give a more complete picture of reality. Other authors undertook a bibliometric study of Creutzfeldt-Jakob disease based on information published in the daily press [19].

Insofar as the research topics were concerned, the need to make the charts explicable rendered it necessary for us to make a general allocation of topics. In this regard, the topics of greatest interest did not experience wide variations across the period reviewed. Among these, special mention must be made of Creutzfeldt-Jakob disease, Scrapie and Chemicals and Drugs. While the relative weight of documents linked to Creutzfeldt-Jakob disease decreased in the second and third versus the first decade, their absolute values increased in the last two decades. A similar pattern was registered by scrapie-related publications, which lost relative weight in the third decade but nevertheless rose in terms of absolute values. Prion-related publications experienced steady growth across the study period, i.e., whereas their percentages barely represented 5.6% in the first decade, they doubled in the second. Nevertheless it was in the third decade that this subject witnessed a boom, becoming the topic with the greatest presence, accounting for 34.3% of all documents published. Something akin happened in the case of BSE: though this topic area only started being reported in the second decade, by the last decade it had come to represent 10.7% of all documents published.

The drastic fall-off in scientific activity in kuru and SVD across the study period, in which publications with a percentage of over 1% appear solely in the first decade, might be related to the waning of the epidemic in the former case and to loss of significance and acceptance of the term in the latter.

Analysis of scientific collaboration among researchers in the three periods into which the study was divided, made it possible to plot its trend, which displays an observable increase in the intensity of collaboration as well as the complexity of the networks formed. Of the clusters that appear in the first decade, only three are very productive, well-defined and maintain important collaboration links, while the remaining clusters show evidence of very few collaborations. In the second decade, though the number of clusters is similar to that of the previous period, the collaboration networks among them nevertheless intensify. In the last period changes take place, both in the collaborations maintained by the researchers and in their numbers. The number of authors rises notably, collaborations among them intensify in great measure, and the number of clusters increases to twelve.

In terms of researchers, each of the periods saw a renewal in the components of the clusters, though some researchers maintained their presence throughout the study period, as was the case of Gajdusek, Brown and Prusiner, who led the most productive clusters. The lack of persistence of collaborations detected in the first and second periods, such as those of Manuelidis E and Manuelidis L or Kimberlin and Walter, is a rare phenomenon compared to the emergence of collaborations, such as those of Tateishi and Kitamoto in the last two, and, above all, the flowering of associations of European researchers in the last period which took place in two well-defined groupings, i.e., the Franco-German association of Zerr, Poser and Hauw, and the Italo-Scottish association of Will, Pocchiari and Ironside.

Bearing in mind that there are several topics of general interest and that these correspond to the most used terms, it might be more instructive to single out those topics in which specific groups have a record of production.

Hence, in the first decade C1 (Gajdusek, Brown and Cathala) tend to publish on epidemiology, whilst C5 (Field, Narang and Shenton) focus more on topics related with kuru and CJD immunology. C3 and C6 devote themselves more specifically to aspects of prion analysis and isolation, and C7 registers greater production in pathology and psychiatry.

The second decade is marked by migration of most clusters towards the centre of the plot, meaning that researchers now share many of the core topics situated in this area. As characteristic topics, C1 works specifically on genetics and pathology, C5 likewise on pathology, C2 devotes itself to prion analysis and genetics, and C4 to genetics. Whereas clusters C3 and C6 specifically publish on Scrapie, C7 publishes on both Scrapie and BSE.

Compared to the first two periods, a more pronounced centripetal effect is in evidence in the last period, along with a wider diversity of topics, over and above those that were of keenest interest to researchers in previous decades. In this decade there is a sharp increase in scientific production on BSE and prion purification, immunology and isolation, on which various clusters -and C1 in particular (Gajdusek, Brown and other authors)- publish. European and Japanese groupings, such as C6 (Hill, Pocchiari and Ironside) and C11 (Kitamoto and Tateishi), surface in CJD-related topics, such as diagnosis, epidemiology, pathology and genetics. Active in topic areas such as prion pharmacology and genetics, are clusters with mutual interest, e.g., C8 (Gambetti, Sy and O'Rourke, among others), C3 (Bugiani, Tagliavini and Salmona) and C9 (Aguzzi, Brandner and Klein). Possible points of coincidence are far more difficult to identify, however, in clusters C2 (Prusiner and other researchers) C7, (Caughey, Priola and Horiuchi), C10 (Hunter, Hope and Goldmann) and C12 (McConnell, Fraser and Taylor), which publish on prion synthesis, inhibition or antagonisms in connection with Scrapie and BSE.

## Conclusion

In brief, this study reveals a high increase in scientific production on prions research, with the establishment of progressive collaboration relationships and a reflection of public health concerns about this problem, dictated by both the extent of the epidemics and the sensitivity of organisations such as the European Union Research Commission.

## Competing interests

The authors declare that they have no competing interests.

## Authors' contributions

ESC participated in study design, data analysis, as well as interpretation of results and he led the writing. CASB participated in data acquisition, and contributed to interpretation of results and manuscript writing. IIM prepared data normalization and participated in data analysis. MRSP prepared data normalization and contributed to manuscript writing. JPC revised biological and public health aspects, data normalization and final version.

## Pre-publication history

The pre-publication history for this paper can be accessed here:



## Supplementary Material

Additional file 1 **Table 4.doc. Topics registering a frequency > 1%, 1973–2002**. Table shows research topics of greatest interest, over the three sub-periods. It shows also absolute and percentage values. Click here for file

Additional file 2 **Table 5.doc. Authors who compose the different clusters, 1973–1982**. Table 5 shows authors' clusters in the first sub-period of the study. Click here for file

Additional file 3 **Table 6.doc. Authors who compose the different clusters, 1983–1992**. Table 6 presents authors' clusters in the second sub-period of the study. Click here for file

Additional file 4 **Table 7.doc. Authors who compose the different clusters, 1993–2002**. Table 7 presents authors' clusters in the final sub-period of the study. Click here for file

## Figures and Tables

**Table 1:** Percentage trend in scientific production with respect to 1973

Year	Documents published	Annual increase (base year 1973)	Year	Documents published	Annual increase (base year 1973)
1973	**78**	100.0%	1988	**125**	160.2%
1974	**59**	75.6%	1989	**113**	144.8%
1975	**66**	84.6%	1990	**148**	189.7%
1976	**62**	79.4%	1991	**213**	273.0%
1977	**62**	79.4%	1992	**202**	258.9%
1978	**65**	83.3%	1993	**246**	315.3%
1979	**61**	78.2%	1994	**277**	355.1%
1980	**77**	98.7%	1995	**236**	302.5%
1981	**81**	103.8%	1996	**360**	461.5%
1982	**74**	94.8%	1997	**407**	521.7%
1983	**80**	102.5%	1998	**415**	532.0%
1984	**93**	119.2%	1999	**492**	630.7%
1985	**114**	146.1%	2000	**528**	676.9%
1986	**144**	184.6%	2001	**650**	833.3%
1987	**116**	148.7%	2002	**680**	871.7%

**Table 2:** Geographical distribution of scientific production. Absolute values and percentages

**1973–1982**			**1983–1992**			**1993–2002**		
Country	No. docs.	%	Country	No. docs.	%	Country	No. docs.	%

USA	211	40.2	USA	463	41.1	USA	1087	26.7
UK	115	21.9	UK	209	18.5	UK	827	20.3
Japan	38	7.2	Japan	115	10.2	France	394	9.7
France	38	7.2	France	57	5.1	Germany	325	7.0
Italy	14	2.7	Germany	55	4.9	Japan	286	7.0
Australia	12	2.3	Italy	43	3.8	Switzerland	187	4.6
Czechoslova-kia	12	2.3	Poland	31	2.8	Italy	155	3.8
Germany	9	1.7	Russia	19	1.7	Spain	74	1.8
Switzerland	9	1.7	Canada	13	1.2	Holland	72	1.8
Canada	8	1.5	Spain	13	1.2	Poland	72	1.8
Poland	7	1.3	Israel	13	1.2	Canada	59	1.5
Chile	6	1.1	Switzerland	12	1.1	Austria	58	1.4
Spain	6	1.1	Others (24)	84	7. 5	Australia	55	1.4
Others(14)	40	7.6		1127	100	Israel	54	1.3
	525	100				Belgium	42	1.0
						Others (38)	319	7.8
							4066	100

**Table 3:** Distribution of entries by type of document

**Document type**	**Absolute frequency**	**Percentage**
Journal Article	6352	81.4%
Letter	774	9.9%
News	404	5.2%
Editorial	206	2.6%
Others	64	0.8%
Total	7800	100%
