# Supplementary material for: Trends in scientific activity addressing transmissible spongiform encephalopathies: a bibliometric study covering the period 1973–2002
Source: BMC Public Health. 2006 Oct 6;6:245. doi: 10.1186/1471-2458-6-245 (PMC1615877; doi:10.1186/1471-2458-6-245)
Supplement: Additional file 1 — Table 4.doc. Topics registering a frequency > 1%, 1973–2002. Table shows research topics of greatest interest, over the three sub-periods. It shows also absolute and percentage values. [file 1471-2458-6-245-S1.doc]

| **1973-1982** | | | **1983-1992** | | | **1993-2002** | | |
| --- | --- | --- | --- | --- | --- | --- | --- | --- |
| **Topics** | **Frequency** | **%** | **Topics** | **Frequency** | **%** | **Topics** | **Frequency** | **%** |
| Nervous system | 154 | 22.5 | Chemicals and Drugs | 435 | 32.3 | Chemicals and Drugs | 1234 | 28.8 |
| Nervous system diseases | 150 | 21.9 | Nervous system | 279 | 20.7 | Biological sciences | 769 | 17.9 |
| Chemicals and Drugs | 116 | 16.9 | Scrapie | 257 | 19.1 | Nervous system | 569 | 13.3 |
| Scrapie | 112 | 16.4 | Nervous system diseases | 255 | 18.9 | Bovine Spongiform Encephalopathy | 458 | 10.7 |
| Diseases | 79 | 11.5 | Diseases | 163 | 12.1 | Diseases | 424 | 9.9 |
| Anatomy | 70 | 10.2 | Biological sciences | 138 | 10.2 | Organisms | 401 | 9.4 |
| Biological sciences | 60 | 8.8 | Anatomy | 102 | 7.6 | Scrapie | 383 | 8.9 |
| Organisms | 57 | 8.3 | Organisms | 95 | 7.1 | Prions>chemistry | 371 | 8.7 |
| Creutzfeldt-Jakob Disease>Diagnosis | 47 | 6.9 | Creutzfeldt-Jakob Disease >Diagnosis | 71 | 5.3 | Nervous system diseases | 365 | 8.5 |
| Creutzfeldt-Jakob Disease >pathology | 36 | 5.3 | Prions>genetics | 62 | 4.6 | Anatomy | 359 | 8.4 |
| Analytical, Diagnostic and Therapeutic Techniques and Equipment | 26 | 3.8 | Creutzfeldt-Jakob Disease >pathology | 61 | 4.5 | Analytical, Diagnostic and Therapeutic Techniques and Equipment | 342 | 7.9 |
| Psychiatry & Psychology | 26 | 3.8 | Analytical, Diagnostic and Therapeutic Techniques and Equipment | 55 | 4.1 | Creutzfeldt-Jakob Disease >Diagnosis | 224 | 5.2 |
| Creutzfeldt-Jakob Disease >complications | 24 | 3.5 | Creutzfeldt-Jakob Disease >epidemiology | 49 | 3.6 | Prions>genetics | 153 | 3.6 |
| Creutzfeldt-Jakob Disease >epidemiology | 16 | 2.3 | Creutzfeldt-Jakob Disease >complications | 35 | 2.6 | Prion Diseases>genetics | 147 | 3.4 |
| Creutzfeldt-Jakob Disease >aetiology | 15 | 2.2 | Bovine Spongiform Encephalopathy | 34 | 2.5 | Prions Diseases>aetiology | 133 | 3.1 |
| Creutzfeldt-Jakob Disease >microbiology | 14 | 2.0 | Prions>analysis | 33 | 2.5 | Prions>analysis | 132 | 3.1 |
| Prions>growth & development | 14 | 2.0 | Prions>immunology | 28 | 2.1 | Creutzfeldt-Jakob Disease >epidemiology | 118 | 2.8 |
| Creutzfeldt-Jakob Disease >immunology | 13 | 1.9 | Creutzfeldt-Jakob Disease >microbiology | 25 | 1.9 | Prions>biosynthesis | 116 | 2.7 |
| Prions>analysis | 12 | 1.8 | Creutzfeldt-Jakob Disease >metabolism | 24 | 1.8 | Prion Diseases>diagnosis | 112 | 2.6 |
| Prions>isolation & purification | 12 | 1.8 | Prions>isolation & purification | 24 | 1.8 | Psychiatry & Psychology | 102 | 2.4 |
| Slow Virus Diseases>microbiology | 12 | 1.8 | Psychiatry & Psychology | 23 | 1.7 | Health Care | 99 | 2.3 |
| Slow Virus Diseases >transmission | 11 | 1.6 | Creutzfeldt-Jakob Disease >aetiology | 18 | 1.3 | Tech.Food.Beverages | 90 | 2.1 |
| Slow Virus Diseases | 10 | 1.5 | Creutzfeldt-Jakob Disease >genetics | 18 | 1.3 | Creutzfeldt-Jakob Disease >genetics | 86 | 2.0 |
| Creutzfeldt-Jakob Disease >diet therapy | 9 | 1.3 | Prions>physiology | 18 | 1.3 | Prion diseases>pathology | 83 | 1.9 |
| Kuru>diagnosis | 9 | 1.3 | Gerstmann-Straussler-Scheinker Disease>genetics | 16 | 1.2 | Physical Sciences | 80 | 1.9 |
| Kuru>immunology | 9 | 1.3 | Prions>growth & development | 14 | 1.0 | Creutzfeldt-Jakob Disease >pathology | 73 | 1.7 |
| Creutzfeldt-Jakob Disease >genetics | 8 | 1.2 |  |  |  | Prion Diseases>epidemiology | 64 | 1.5 |
| Creutzfeldt-Jakob Disease >diagnosis | 7 | 1.0 |  |  |  | Creutzfeldt-Jakob Disease >aetiology | 62 | 1.4 |
| Kuru>epidemiology | 7 | 1.0 |  |  |  | Creutzfeldt-Jakob Disease >cerebrospinal fluid | 62 | 1.4 |
| Scrapie>pathology | 7 | 1.0 |  |  |  | Creutzfeldt-Jakob Disease >metabolism | 57 | 1.3 |
| Slow Virus Diseases >pathology | 7 | 1.0 |  |  |  | Prions>metabolism | 56 | 1.3 |
|  |  |  |  |  |  | Information science | 54 | 1.3 |
|  |  |  |  |  |  | Prion diseases>metabolism | 53 | 1.2 |
|  |  |  |  |  |  | Creutzfeldt-Jakob Disease >complications | 51 | 1.2 |
|  |  |  |  |  |  | Prions>immunology | 50 | 1.2 |
