# Supplementary material for: Trends in scientific activity addressing transmissible spongiform encephalopathies: a bibliometric study covering the period 1973–2002
Source: BMC Public Health. 2006 Oct 6;6:245. doi: 10.1186/1471-2458-6-245 (PMC1615877; doi:10.1186/1471-2458-6-245)
Supplement: Additional file 2 — Table 5.doc. Authors who compose the different clusters, 1973–1982. Table 5 shows authors' clusters in the first sub-period of the study. [file 1471-2458-6-245-S2.doc]

| **1973-1982** |  |  |  |  |  |  |  |
| --- | --- | --- | --- | --- | --- | --- | --- |
| C1 | C2 | C3 | C4 | C5 | C6 | C7 | C8 |
| GajdusekDC | KimberlinRH | PrusinerSB | FraserH | FieldEJ | HadlowWJ | ManuelidisEE | BertJ |
| BrownP | MillsonGC | CochranSP | DickinsonAG | NarangHK | EklundCM | ManuelidisL | TamaletJ |
| CathalaF | MarshRF | GrothDF | OutramGW | ShentonBK | RaceRE |  |  |
| GibbsCJ Jr | HunterGD | McKinleyMP |  |  |  |  |  |
| AsherDM | CollisSC | BaringerJR |  |  |  |  |  |
| CourtL | HansonRP |  |  |  |  |  |  |
| MastersCL | WalkerCA |  |  |  |  |  |  |
| MoreauDuboisMC |  |  |  |  |  |  |  |
| RohwerRG |  |  |  |  |  |  |  |
