# Supplementary material for: Trends in scientific activity addressing transmissible spongiform encephalopathies: a bibliometric study covering the period 1973–2002
Source: BMC Public Health. 2006 Oct 6;6:245. doi: 10.1186/1471-2458-6-245 (PMC1615877; doi:10.1186/1471-2458-6-245)
Supplement: Additional file 3 — Table 6.doc. Authors who compose the different clusters, 1983–1992. Table 6 presents authors' clusters in the second sub-period of the study. [file 1471-2458-6-245-S3.doc]

| **1983-1992** |  |  |  |  |  |  |  |
| --- | --- | --- | --- | --- | --- | --- | --- |
| C1 | C2 | C3 | C4 | C5 | C6 | C7 | C8 |
| GajdusekDC | PrusinerSB | CarpRI | HopeJ | TateishiJ | BruceME | ManuelidisEE | CaugheyB |
| GibbsCJ Jr | BendheimPE | DiringerH | FosterJD | KitamotoT | FraserH | ManuelidisL | RaceRE |
| CathalaF | DeArmondSJ | KascsakRJ | DickinsonAG | DohuraK |  |  |  |
| BrownP | McKinleyMP | KimberlinRH | HunterN |  |  |  |  |
| ChatelainJ | BoltonDC | WisniewskiHM | MarshRF |  |  |  |  |
| AsherDM | WestawayD | RubensteinR | SomervilleRA |  |  |  |  |
| GoldfarbLG |  | WalkerCA |  |  |  |  |  |
| LiberskiPP |  | MerzPA |  |  |  |  |  |
| PocchiariM |  |  |  |  |  |  |  |
| YanagiharaR |  |  |  |  |  |  |  |
