# Supplementary material for: Trends in scientific activity addressing transmissible spongiform encephalopathies: a bibliometric study covering the period 1973–2002
Source: BMC Public Health. 2006 Oct 6;6:245. doi: 10.1186/1471-2458-6-245 (PMC1615877; doi:10.1186/1471-2458-6-245)
Supplement: Additional file 4 — Table 7.doc. Authors who compose the different clusters, 1993–2002. Table 7 presents authors' clusters in the final sub-period of the study. [file 1471-2458-6-245-S4.doc]

| **1993-2002** |  |  |  |  |  |
| --- | --- | --- | --- | --- | --- |
| C1 | C2 | C3 | C4 | C5 | C6 |
| BrownP | PrusinerSB | BugianiO | ZerrI | LaplancheJL | WillRG |
| LiberskiPP | CohenFE | TagliaviniF | KretzschmarHA | HauwJJ | PocchiariM |
| GajdusekDC | DeArmondSJ | SalmonaM | GroschupMH | DormontD | IronsideJW |
| RubensteinR | GabizonR | GhettiB | PoserS | DeslysJP | ZeidlerM |
| CervenakovaL | GrothD | HarrisDA | WindlO | DelasnerieLaupretreN | |
| JeffreyM | BaldwinMA | GiacconeG | BrownDR | BrandelJP |  |
| WellsGA | WestawayD | LehmannS |  |  |  |
| BudkaH |  | ForloniG |  |  |  |
| GoldfarbLG |  | CollingeJ |  |  |  |
| MastersCL |  |  |  |  |  |
| CarpRI |  |  |  |  |  |
| BruceME |  |  |  |  |  |
| WilesmithJW |  |  |  |  |  |
| C7 | C8 | C9 | C10 | C11 | C12 |
| CaugheyB | GambettiP | AguzziA | HunterN | KitamotoT | McConnellI |
| PriolaSA | SyMS | BrandnerS | HopeJ | TateishiJ | FraserH |
| HoriuchiM | O'RourkeKI | KleinMA | GoldmannW |  | TaylorDM |
| LindquistS | ParchiP | WeissmannC |  |  |  |
| ChesebroB | PetersenRB |  |  |  |  |
|  | CapellariS |  |  |  |  |
|  | WongBS |  |  |  |  |
